# Labels, cognomes, and cyclic computation: an ethological perspective

**DOI:** 10.3389/fpsyg.2015.00715

**Published:** 2015-06-03

**Authors:** Elliot Murphy

**Affiliations:** Division of Psychology and Language Sciences, University College London, London, UK

**Keywords:** minimalism, labeling effects, cognome, animal cognition, formal language theory, language evolution

## Abstract

For the past two decades, it has widely been assumed by linguists that there is a single computational operation, Merge, which is unique to language, distinguishing it from other cognitive domains. The intention of this paper is to progress the discussion of language evolution in two ways: (i) survey what the ethological record reveals about the uniqueness of the human computational system, and (ii) explore how syntactic theories account for what ethology may determine to be human-specific. It is shown that the operation Label, not Merge, constitutes the evolutionary novelty which distinguishes human language from non-human computational systems; a proposal lending weight to a Weak Continuity Hypothesis and leading to the formation of what is termed Computational Ethology. Some directions for future ethological research are suggested.

The assumption of many contemporary linguists is that part of the complexity of the world’s languages is encoded in the human computational system. In one of the most prominent branches of linguistics, the Minimalist Program (Chomsky, 1995), the operation which language’s human-unique aspects may reduce to is termed “Merge.” This constructs a new syntactic object out of two already formed. Merge(*the*, *man*) would form {the, man}. Assimilating standard accounts (Chomsky, 2013; Boeckx, 2014), we can define the operation as follows:

(1) Merge

Select two lexical items α and β and form the set {α,β}:

**M**(α,β) = {α,β}

Merge is a computational operation in the traditional sense that it is being described at a higher level of abstraction than algorithmic procedures and the implementational level of neurons and dendrites (Marr, 1982). Early minimalism held that when Merge targets two syntactic objects, α and β, forming a new object, Γ, the label of Γ is either α or β. That is, when two lexical items (LIs) are merged, one of them “wins” and is projected as the head: **M**(α,β) = {α{α,β}} or {β{α,β}}. With *red car*, the label is *car* since this word determines the category of the phrase (a noun phrase, not an adjectival phrase). Narita (2011, p. 191) summarizes this “old intuition” by writing that “it is simply an ordinary fact about language that “noun phrases” are interpreted in a “nouny” way.” The label indicates the structure’s meaning to the conceptual-intentional (CI) system (an axiom assumed in Chomsky, 2013; Epstein et al., 2014; and much other work). To put it plainly, labeling is the operation which chooses which lexical features select the phrasal category. The central arguments of this paper will be that, firstly, this form of asymmetric hierarchy is created by labeling, and secondly, that this process is unique to humans. To briefly illustrate the latter point, even though it has been well established that birds can “chunk” song units, these do not appear to contain the properties out of which the chunks are composed. The *warble-rattle* chunks of chaffinches (Riebel and Slater, 2003), for instance, do not host the properties of either of the individual song units. There are no “warble phrases” or “rattle phrases,” just warbles and rattles externalized in a particular sequence.

A recent study of relational nominals (Adger, 2013) holds that labeling is exocentric, separating it from the merging and moving seen in standard minimalist accounts. Adger (2013) is troubled by the relegation of labeling to a mere side effect of Merge in Bare Phrase Structure, a strand of minimalist syntax in which LIs are Merged to check features before being transferred to the CI and sensorimotor (SM) systems. Finally, Chomsky claims that the label of a structure is selected through an epiphenomenal process of computational efficiency, a reflex of natural law (Chomsky, 2013, pp. 43–46, Boeckx, 2009, pp. 52–54, see Brody, 1998 for an alternative account). Label-free systems relying purely on set-formation and feature-checking of the kind developed by Collins (2002) and Narita (2012) are in some respects theoretically elegant, but they deal poorly with movement, since they “effectively predict that a DP in the specifier of another DP will always be more prominent for syntactic relations outside the latter DP” (Adger, 2013, p. 16). Labels are useful for showing how dominance relations are “exploited” by the grammar (Hornstein, 2009, p. 46), and they can also derive certain θ-Criterion effects (Narita, 2009). Tomalin (2007, p. 1784) observes in his account of the development of the theory of recursive functions that “even if a label-free system is proposed, the essential constructional process remains the same”; that is, set-formation and labeling are essential for the construction of “a potentially infinite set of hierarchical structures.” Label-free systems also ignore the extra-linguistic simplicity of more atomic accounts which differentiate between the distinct cognitive operations of what Poeppel (2012) has named in general the “human cognome” (the finite set of operations available to the human brain) and Balari and Lorenzo (2013) in relation to the language faculty the “Central Computational Complex” (CCC; the finite set of operations available to the nervous system). These syntactic perspectives will be discussed below, serving as the basis from which to assess broader questions in the life sciences.

The concerns of linguists also bear directly on Tinbergen’s (1963) seminal ethological research program, which aimed to explore development (ontogenesis), how a behavior develops in an organism, and evolutionary history (phylogenesis), how it developed in the species. By following his “aims and methods,” what ethology has revealed about the cognition of non-human animals has been highly instructive. Yet as I intend to show, neither the general proposals of ethology, nor its specific suggestions concerning the necessity of interdisciplinary collaboration have been taken seriously by substantial elements of the language sciences. Instead, the observation that language has no biological equivalent from which direct comparative study could proceed has been used to discourage comparative biological and computational inquiry. The goal of this contribution is to suggest new methods that these and related fields can use to investigate the nature of language and the brains of both humans and non-humans.

One of the central claims of what follows is that the ethological implications of decomposing Merge into set-formation (Concatenate) and Label (see “Simplest Merge and Labeling”) have not been appreciated by researchers of non-human cognition (and, as later sections detail, researchers of human cognition as well). The ethological background will be outlined in more concrete terms in Section “Approaching Computational Ethology: The Evidence,” concrete terms shortly, and once we view the matter from the perspective of what I will call “Computational Ethology,” which investigates comparatively the cognomes of non-humans, surprising theoretical consequences arise. The section “Simplest Merge and Labeling” discusses syntactic primitives within a minimalist framework. The section “Approaching Computational Ethology: The Evidence” presents the bulk of ethological evidence we will discuss. Those aspects of the cognome deemed human-unique in Section “Approaching Computational Ethology: The Evidence” deemed human-unique are discussed in Section “Homo Combinans or Homo Projectans?” Finally, the last section suggests some new directions for testing the central hypotheses put forward. While Hauser et al. (2002, 2014) used primate calls to draw attention to the supposed human-uniqueness of Merge, I will instead focus primarily on birdsong as evidence for the human-uniqueness of Label. Part of this paper will be dedicated to an ethological survey, but in order to know what it is we are searching for a theoretical syntactic backdrop is required. The following pages will consequently be divided into between ethological and syntactic explorations, supplemented by evolutionary, cognitive and other perspectives where appropriate.

## Simplest Merge and Labeling

This section presents an introductory discussion of the concepts relevant to our ethological discussion (and some which explicitly will not be), a necessary step in placing syntactic inquiry within the life sciences.

### Concatenation

Merge is plainly a core operation of the computational system of human language, but its scope remains unclear. A dominant view in generative grammar is that word movement is a primitive operation, a case of “just simple Merge” for (Chomsky, 2012a, p. 3; Chomsky, 2013, p. 40). There is sound evidence, however, that Merge is actually a form of Concatenation plus Label (Hornstein, 2009). Hornstein (2009, p 113), for instance, note that the operation combine(a,b) is not primitive, and consists of LABEL[CONCATENATE(A,B)]. These investigations are central to what I have elsewhere called the Decompositionalist Project (Murphy, 2015c), which explores the fine structure of the cognome through embracing the full range of interdisciplinary investigations in the cognitive and biological sciences. Concatenation takes two objects and forms from them an unordered set, {α β}. This is a case of what Epstein et al. (2014, p. 471) term “Simplest Merge,” noting that “composite operations are in general unwelcome as we search for the primitive, minimal, undecomposable operations of NS [narrow syntax].” Labeling then imposes order after searching for a head, say β. Further, the “copy” of β, <β>, would be left after β has been concatenated in a new position: {_β_ α β} {_β_ β {_β_ α <β>}}. In the simplest cases, this would represent certain types of question formation, where a copy of the *wh*-phrase is left in its initially concatenated position, for reasons of interpretability at the CI interface: “What did you buy <what> ?” The implications of assuming unordered set-formation to involve Concatenate, and not Merge, will become clear in the next section. In addition, for ease of ethological exposition below I will put aside the standard context-free definition of Concatenation involving conjoined, ordered sets.

### Agree

Consider the sentences in (2):

(2) a. There *seems* to be likely to be *a man* in the garden.

b. There *seem* to be likely to be *men* in the garden.

As noted in the minimalist literature, in such *there*-expletive constructions the main verb exhibits long-distance number agreement with an associate noun phrase. This featural covariation cannot be delivered by pure set-formation, and so an independent operation, Agree, has often been appealed to (Chomsky, 2000a), in which a *probe* with an unvalued feature searches its domain for an eligible *goal* with a valued feature to match (Narita, 2014, p. 31). The value of the goal is then copied onto the probe, establishing syntactic covariance between features of two different objects. But for the purposes of comprehensiveness it is worth noting that, unlike Concatenate and Label, Agree is not required at all for reasons of economy and there exist at least “three different ways of modeling long distance agreement phenomena” without it (Hornstein, 2009, p. 117). I will assume with Kobele (2006, p. 143) that Agree is just movement “with particular interface effects” (contra Narita, 2009), with the lower copy being pronounced, although standard formulations of Agree crucially impose no demands on the interfaces, being what Adger and Svenonius (2011) dub a “syntax-internal” operation (see also Arregi and Nevins, 2013).

### Natural Numbers

If Chomsky (2012b) and Hinzen (2009) are right that Merge yielded the natural numbers via the successor function, then it must be free of “triggers” such as the valuation of features, as often assumed, since the natural numbers clearly do not rely on φ-features. They also do not appear to require labeling, as Tomalin (2007, p. 1795) writes: “[T]he objects generated by the repeated application of Merge are not associated with labels … and therefore the computational processes of arithmetic do not seem to require the same information concerning hierarchical structure that is required by the computational procedures that generate syntactic objects.” This freeing of Concatenate from lexical influence will have implications for our ethological account.

### Adjuncts

Concatenation has gone by many names: “Adjoin” (Adger, 2003), “Adjunction” (Hinzen, 2011), “Concatenate” (Pietroski, 2002). Abstracting away from the particular concerns of these papers, each definition assumes that the operation purely involves unordered set-formation. If Concatenate is not unique to humans, the sentential structures formed by may effectively be residues of ape cognition, as suggested by Boeckx (2008). Chomsky (2001b) gives adjunction a “principled explanation” by saying that the CI systems need an operation of predicate composition, but this amounts to a functional requirement which serves only to rationalize the existence of a computational operation. Not only does this functionalist perspective fly in the face of naturalism (Chomsky’s, 2000b), this kind of argument should not be welcomed given our lack of knowledge as to what the CI system actually consists of (see Hinzen, 2009). On this occasion, the ethological viewpoint Boeckx promotes sheds more light on the puzzle of adjunction than other minimalist accounts. The features of adjuncts suggest that they amount to the least central structures syntax can produce, hence why Chomsky (2001b) points out that when α adjoins (concatenates) to β, β behaves as if α was “not there, apart from semantic interpretation.” Adjuncts do not participate in control or feature-checking and are not selected, and if Hinzen (2009) is right that semantic objects are creatures of syntax, then since Concatenate is a simpler computational operation than Merge, we would expect its semantics to be less rich. This prediction seems to hold true: Neo-Davidsonians like Pietroski (2002, 2005) assume that right-Merged adverbials have a very simple, but still compositional conjunctive semantics. Their interpretation consists of conjoining predicates. Intuitively, *walk quickly* means *there is a walking that is quick*:

(3) *walk quickly*: (∃e) e is a walking and e is quick

Arguments lack this semantics: *Jason ran* does not mean *there is a running and it is Jason*, but rather: ∃e [ran (e) and THEME (Jason, e)]. Labeling can be appealed to in order to deliver this outcome (Gallego, 2010, p. 16, Hornstein and Pietroski, 2009, p. 117, Pietroski, 2008), since it produces a result which cannot be reduced to the meaning of its parts. Labeling mismatches “invoke thematic concepts,” for Hornstein (2009, p. 125). Adjunction is thus blind to “participant-of” relations, and we would expect any species only capable of concatenation to be unable to compute “thoughts” like *Jason ran*. This has led some to speculate that simian cognition can process modificational structures like “(the) [red [heavy [box]]].” Hence non-hierarchical adjuncts come “for free” from our simian ancestors through this set-formation operation, while argument structure requires a labeling algorithm to yield v*Ps.

### Labeling

Hornstein (2009), Boeckx (2010), Adger (2013), and various others have suggested that the headedness/endocentricity generated by labeling (though which one element determines the identity of the larger phrase) is a property unique to language distinguishing it from other modes of hierarchical cognition (e.g., kinship relations). Many labeling theories, of the kind seen in Boeckx (2008), propose that it is lexical properties which determine an LI’s capacity for specifiers and complements. Chomsky (2013, 2015b) likewise proposes that head detection can be reduced purely to minimal search of an LI, while labeling must take place at the point of transfer to the interfaces (Chomsky, 2015b), obeying principles of least effort and other “third factor” constraints (Chomsky, 2005 et seq.; Narita, 2012, p. 156) terms this “minimal head detection.” The notion of prominence here refers to those aspects of an LI which allow the labeling algorithm to identify it as an atom to be headed (Narita, 2011, 2012, pp. 190).

(4) Minimal head detection

For any syntactic object Σ, the head of Σ is the most prominent LI within Σ.

But these approaches are too lexico-centric, smuggling in a further computation (call it “Inspect”) when labeling an {XP, YP} structure, inspecting both heads of these constituents to determine which should be labeled. More worryingly, Chomsky’s (2013, 2015b) labeling algorithm, which relies on the notion that word movement yields an asymmetry permitting labeling, simply restates the pre-minimalist argument that a phrasal head projects but does not explain why it projects.

### The Cognome

Here we must introduce further operations if our ethological discussion is to have any context. The operation Transfer sends the syntactic object taken from the lexicon by Select and constructed by Concatenate and Label to the interfaces. Since recent studies suggest that the timing of Transfer to the two interfaces differs (Marušič, 2009), this has been divided into two operations: Spell-Out at SM (Uriagereka, 2012) and Interpret at CI (Lasnik and Uriagereka, 2005, p. 240). The former process is commonly termed externalization, prompting Berwick et al. (2013, p. 91) to call interpretation at CI “internalization.” Feature-checking and copying, Hornstein (2009, p. 3) notes, are “almost certainly operative in other cognitive domains, albeit with different expressions being copied and different features being checked.” Given this, the human cognome, which we can notate as ς_HUMAN_ (ς_H_), amounts to the following operations:

(5) ς_HUMAN_ = *ω*(SELECT), κ(CONCATENATE), χ(COPY), *ω*(CHECK), ξ(LABEL), *ψ*(INTERPRET), *δ*(SPELL-OUT)

The remainder of this paper will review the ethological literature before suggesting how this computational model could be applied to some of the most thoroughly researched animals.

## Approaching Computational Ethology: The Evidence

### Formal Language Theory

**FIGURE 1:**
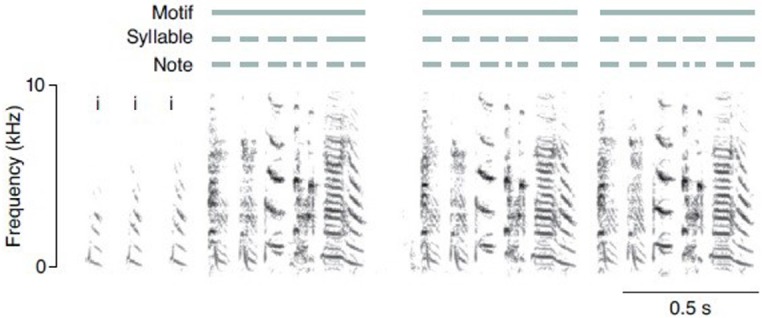
**Sound spectrum of Zebra finch song with hierarchical structure (from Berwick et al., 2011, p. 114)**.

Beginning with the most well studied organisms, it has been shown that the birdsong notes of Zebra finches often combine into multi-element syllables, which combine into motifs, which in turn string together into song bouts (Cynx, 1990); a process depicted in Figure 1.

These song bouts exhibit hierarchical structure, but there is no labeling, no mapping of complexes into lexical equivalence classes as is seen in human language. We can interpret these findings from the perspective of the traditional hierarchy of formal languages (see Jäger and Rogers, 2012 for an introduction):

(6) *Chomsky Hierarchy* (Chomsky, 1956b, 1957, 1963):

Type 0: Unrestricted systems (Turing machine)

Type 1: Context-sensitive systems (linear-bounded automaton; sets of sets of symbol sequences)

Type 2: Context-free systems (push-down automaton; sets of symbol sequences)

Type 3: Regular systems (finite-state automaton; symbol sequences)

**FIGURE 2:**
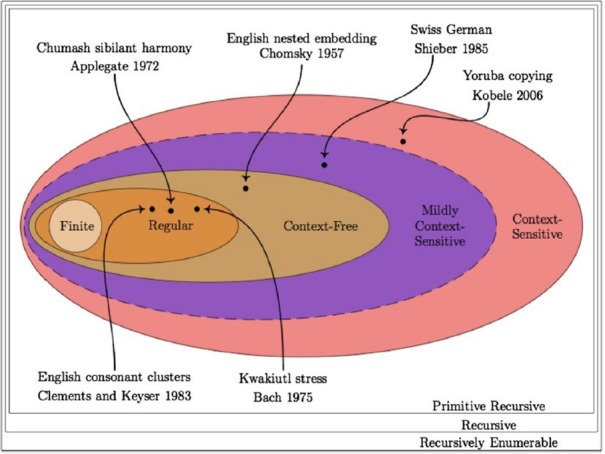
**The Chomsky Hierarchy and corresponding natural language patterns (from Heinz, forthcoming)**.

I should note from the outset that particular limitations of this hierarchy will be discussed below, but due in Section “Computational Ethology,” but due to the vast majority of ethological work informed by linguistics being center on formal language theory, it is necessary to initially discuss this work within the standard framework. Formal language theory can indeed be a useful tool in exploring the computational resources of and patterns from different cognitive domains. For instance, Type 2 context-free phrase structure grammars were proven insufficient for human language in the 1980s (Huybrechts, 1984; Shieber, 1985). Human language is thought to lie “beyond” Type 2 as mildly context-sensitive (MCS) but “below” Type 1 languages (Joshi, 1985), which cannot be parsed in polynomial time. MCS languages are distinguished from Type 3 languages by their narrow number of overlapping dependencies and their ability to nest clauses inside clauses of indiscriminate depth. Phrase structure grammars assume a counting mechanism, implemented by push-down stack memory, and so can generate A^n^B^n^ structures of the kind A^i^B^i^ *i* ≤ 4, a finite subset of this language in which *n* is no greater than 4. Finite-state grammars cannot achieve this (Chomsky, 1956a), although they can generate (AB)^n^ languages. A^n^B^n^ languages are thus not necessarily Type 3, but can make use of Type 2 computations. Type 1 and 2 grammars require a working memory space, dealing with dependencies between constituents. Song sequences are usually non-random but are also non-deterministic, with the probability of a song type depending on one or more preceding type (Catchpole and Slater, 2008, pp. 208–209). If dependents are further back than the immediately preceding type, then a higher order Markov chain is required, not a context-free grammar. A representation of the power of formal languages is found in Figure 2.

European starlings (*Sturnus vulgaris*) can distinguish A^n^B^n^ auditory sequences from those with an (AB)^n^ structure according to an influential study by Gentner et al. (2006), while nightingales can sing motifs with notes embedded within looped chunks (Todt and Hultsch, 1998). An (AB)^n^ structure is a case of a regular/finite-state grammar and is in the Strictly Local class SLk, being constructed from a finite alphabet with a beginning ( × ) and end ( × ). (AB)^n^ is an SL2 stringset definable by a set of 2-factors (Rogers and Hauser, 2010, pp. 9–10): D(AB)n = { × A,AB,BA,B × }. Set-formation and the atomisation of call units is displayed, then, but a labeling operation is absent. Call unit α can be concatenated with unit β, forming the linear sequence <α, β> , but this structure is never given an independent computational identity. Abe and Watanabe (2011, p. 1070) claim that finches can distinguish syllable strings constructed by context-free grammars, appearing to be sensitive to centre-embedded structures. Note that embedding and recursion are not the same (Fitch, 2010b; Watumull et al., 2014a,b), although memory constraints permit and encourage recursion.

It should also be noted, however, that mastering A^n^B^n^ structures involves comparing two sequences of elements, a task achievable by sound short term memory and not necessarily Type 2 cognomes (see Ojima and Okanoya, 2014, pp. 166–170 for critical commentary). MCS languages also go beyond this by generating A^n^B^m^C^n^D^m^ stringsets with cross-serial dependencies; no human language is known to require further power. Consequently, any ethological model which attempts to show the context-freeness of non-human syntax needs to demonstrate that the As and Bs are paired together. Merely showing that the number of distinct elements are equal, as in the Gentner et al. (2006) model, does not entail a dependency. Fitch and Friederici (2012, p. 1943) document that counting and comparing across phrases, the computation required to recognize A^n^B^n^, is “difficult or impossible for most tested non-human species.” They also suggest that the concept of bilateral/mirror symmetry requires a context-free grammar capable of recognizing A^n^B^n^ structures and, as a result, able to engage in mirror symmetry detection (Fitch and Friederici, 2012, pp. 1943–1944). A^n^B^n^ languages, though context-free, can still be recognized by a finite-state automata augmented with a simple counter (Jäger and Rogers, 2012, p. 1962, Zimmerer et al., 2014), bringing into question what computational capacities ethologists are in fact investigating. As Zuidema (2013a) observes, Gentner et al. (2006) also presented stimuli to teach starlings to distinguish A^n^B^n^ from (AB)^n^, but no stimuli was presented which would allow them to exclude A^n^B^m^, leading to ambiguities over whether the starlings could actually learn Type 2 languages.

A further problem with many experiments (e.g., Abe and Watanabe, 2011; Ten Cate and Okanoya, 2012) is their lack of specificity about the object of investigation (and, in the case of Abe and Watanabe, 2011, their additional mistakes in experimental materials renders their findings extremely suspect (see Berwick et al., 2012)), demonstrating a general failure to distinguish between the ability to implement, learn, and have a preference for a Type 2 language. To this day, the MCS of non-humans has not been tested, with ethologists typically favoring the search for identification of nesting. Even the most recent research (Kershenbaum et al., 2014) suggests only that non-Markovian dynamics like the “renewal process” (a strong tendency to repeat elements) may characterize the vocalizations of seven taxa including Bengalese finches, rock hyraxes and killer whales, rather than Markovian processes or, indeed, context-free grammars. Further research is needed to determine whether birds can recognize Type 2 languages and which family of Type 2 or 3 languages matches their auditory cognition; for instance, can finches recognize a more complex finite-state pattern such as A^1^(BA^1^)^1^, where 1 indicates paired elements?

For now it at least seems clear that, lacking a lexicon or identifiable semantics (Catchpole and Slater, 2008), birdsong is best characterized as “phonological syntax” (Marler, 1998), resembling most strongly human sound structure. Although human phonological structure is characterisable via finite-state machines, there the similarities appear to end with birdsong syntax. Gentner and Hulse (1998), for instance, proved that a first-order Markov model serves to describe the majority of starling motif sequences, with every motif being predictable by its immediate predecessor.

From an evolutionary-developmental (evo-devo) perspective, Balari and Lorenzo (2013) reinterpret the Chomsky Hierarchy as a family of Owenian archetypes (CompuT1, etc.). While we may not have a comprehensive understanding of the morphological properties of these phenotypes, since the early days of generative grammar it has been possible to develop comprehensive computational characterisations of them. Moving from one computype to a less complex one is not prevented, something which may be analogous to “the putative episodes in brain reduction proposed for some metazoan lineages” (Balari and Lorenzo, 2013, p. 125). Type 2 grammars are scarce in nature (Hurford, 2011), so a shift from Type 3 to MCS giving rise to human grammar is more likely to have occurred than a Type 2-MCS shift—an observation quite apart from the fact that both these languages can be generated by an MCS grammar.

### Hierarchy

As mentioned, the songs of Bengalese finches are markedly predictable in that each sequence is constructed purely from concatenating a new sub-sequence to the end of another. This constitutes a form of trivial tail recursion (Fitch, 2010a). Bird song, whale song and gibbon song are thus supposedly cases of “phonological syntax,” but not “lexical syntax.” Bengalese finches are also capable of “segmentation and chunking,” basic processes thought to be involved in human language acquisition (Takahasi et al., 2010, p. 481). As with phonological syntax, labeling is not necessary for hierarchy. There is no endocentric labeling in syllables which have a nested [*σ*[onset] [*ρ* [nucleus] [coda]]] structure; further, there is no repeated nesting (syllables within syllables). It has relatedly been claimed that wild mountain gorillas appear to prepare nettles for eating in a hierarchical procedure (Hurford, 2011), free of endocentricity:

(7) Nettle preparation (adapted from Byrne and Russon, 1998): Find patch→Collect leaf-blades; Enough? ↺ →[Strip stem; Enough? ↺ →Tear-off petioles]→Clean→Fold blades. [Eat nettles]

Byrne and Russon (1998, p. 667) conclude that “great apes suffer from a stricter capacity limit than humans in the hierarchical depth of planning.” Indeed, as Fitch and Friederici (2012, p. 1936) comment, “as our understanding of neural computation in vertebrates progresses, it seems likely that different hierarchies will arise.” Pulvermüller (2014) even suggests that locality conditions in syntax may impose constraints on the “syntax of actions” and motor planning (see also Moro, 2014 for objections, and Boeckx, 2014 for a review of the Pulvermüller-Moro exchange). Be that as it may, what appears to be hierarchical may simply be automated, with the brains of wild mountain gorillas possibly having created a looped routine characterisable in Markovian terms (see Penn et al., 2008, p. 117 for criticisms of attributing hierarchical forms of cognition to non-humans).

### Compositionality

Returning to issues of combinatorics, free-ranging male Campbell’s monkeys have been shown to respond to disturbances with around twenty loud calls and six call types: *krak*, *krak-oo*, *hok*, *hok-oo*, *wak-oo*, and *boom* (Ouattara et al., 2009). The majority of sequences include a series of *krak-oo* calls which are occasionally concatenated with other types, while the tempo is modulated by the sense of emergency. The contact calls of female Campbell’s monkeys can be externalized as single units (ST1, SH2 etc.), combined, e.g., Concatenate(ST1, SH2) = {CT}, or distinguished by a suffix (e.g., Concatenate(SH2, frequency-modulated-arch) = {CH6}. These sequences are used to communicate narrow contextual information, the content of which can be strongly modified by slight changes (Lemasson et al., 2013).

The computational capacities required for such concatenation do not seem to extend beyond Type 3 systems, and it appears that the monkeys are restricted to a single application of concatenation. Schlenker et al. (2014) have developed a formal semantic analysis of the calls of free-ranging Campbell’s monkeys, proposing that *krak* and *hok* are “roots” which independently convey information (and can have attributed to them a propositional semantics, “type t”) and which can be optionally affixed with *-oo*, whilst calls which begin with *boom boom* indicate a non-predatory context. *Krak* appears to have different “lexical entries” (conceptual content) for two different groups of monkeys in Tai forest (Ivory Coast) and on Tiwai island (Sierra Leone), and so seems to be “underspecified” in the way that many human language constructions require pragmatic *strengthening* and *saturation*. These call combinations can be generated by a finite state grammar (Schlenker et al., 2014, p. 454), with a “leopard call,” for instance, having the following structure (“*” = multiple occurrences, “K” = *krak-oo*, “k” = *krak*):

(8) Leopard call: k K*, k k*

The cognome of white-handed gibbons also permits the concatenation of a finite set of units to yield duet and predator songs (Clarke et al., 2006), utilizing combinatorial rules to advertise pair bonds and repel conspecific intruders. Numerous monkey species also produce call combinations which convey complex meanings distinct from the atomic calls, but do this in a highly constrained and non-cyclic fashion (Zuberbühler, 2012). Bottlenose dolphins also demonstrate “a capability for reasoning about higher order relations through the spontaneous combination or concatenation of previously generalized concepts” (Herman et al., 2008, p. 139). To stress the central argument of this paper, as with birdsong a labeling operation appears to be absent from all of these computations.

We can conclude from this section that the evidence in favor of non-human MCS grammars is either non-existent or based on speculation and misrepresentation, while the evidence for Type 2 grammars is dubious, although this may be due more to the methodological flaws of a young field (Fitch and Hauser, 2004; being the first major study) than the computational properties of its objects of inquiry. The next section will explore the implications of this in relation to emerging developments in minimalist syntax.

## Homo Combinans or Homo Projectans?

The core suggestion of mainstream minimalism that the evolution of grammar reduces to the appearance of Merge after a chance mutation (Chomsky, 2010) leaves us with the risk that genericity of computation may block insight into novelty of syntactic structure (see Boeckx, 2015 for comments on the relationship between minimalism and biolinguistics). In Berwick (2011) view, along with the lexicon Merge is “all you need” for language evolution [which may be “the hardest problem in science” (Christiansen and Kirby, 2003)], recently updated in his integration hypothesis (IH; Miyagawa et al., 2014), discussed below. Minimalism has, I believe, identified “a new aspect of the world” (Mukherji, 2010, p. 27), a new joint of nature, though it is not to be found in concatenation, but labeling.

### Symmetry Breaking and Cyclic Computation

This section explores the implications of assuming what I will call the *Labeling Hypothesis*:

(9) Labeling Hypothesis:

The operation Label constitutes the evolutionary novelty which distinguishes the human cognome from non-human cognomes.

The novelty of adopting this hypothesis within an ethological framework of assumptions becomes particularly vivid when we note that even notable minimalists often sideline labeling, with Berwick et al’s (2011, p. 119) review of avian and human novel computational capacities merely noting on a single occasion that “Birdsong motifs lack word-centric “heads” and so cannot be individuated via some internal labeling mechanism to participate in the construction of arbitrary-depth structures.” This observation is accurate, but its implications for the evolution of grammar are never discussed, nor are appropriate connections drawn between the findings of ethologists and syntacticians. Berwick et al., (2011, p. 120) further speculate that, “with the addition of words, humans acquired the ability to label and “hold in memory” in separate locations distinct phrases such as *Allison ate apples*.” But this lexico-centrism gets the causation backward, and ignores the possibly syntax-independent nature of labeling. The arrival of Label (ξ) would have allowed the construction of a lexicon, transferring the same atoms of computation (roots) in different categorically specified structures, e.g., _N_√*walk* or _V_√*walk*. The present model thus departs from Berwick et al.’s account of human computational complexity and non-human bounded call systems, since an anti-lexicalist focus on the operation ξ permits a finer-grained hypothesis concerning the cognitive (and, ultimately, neurobiological) capacities of humans.

This brings us to the conclusions drawn in Boeckx’s (2014, p. xii) recent study, the central thesis of which is that “it is the lexicon that depends on syntax and not the other way around.” The asymmetries found in language such as the external/internal argument distinction and the binder-bindee relation rely on c-command, whose asymmetry emerges from labeling. Boeckx (2014, p. 38) attempts to derive labeling effects and all linguistic asymmetries from cyclic transfer, a worthwhile project to undertake, as Marantz’s (2007) suggestion that roots are categorized at the “phase” level (that is, at the point of transfer, typically assumed to be DP, v*P and CP, but see Murphy, 2015b) would suggest. But given the possibly language-independent (and CI/SM-independent) nature of labeling, I suggest that a separate computational procedure is required, not just a freeing of concepts from their selectional restrictions as Boeckx argues for. This permits us to explain certain lexical content in terms of labeling choices; a *red ball* is an object (NP/DP), not a property (AP), and *John ran* is an event (*v*P), not a special kind of thing (NP/DP). ξ is required if only because there is nothing in the set-theoretic definition of Merge which leads to symmetry-breaking. This is a primary motivation behind Hornstein and Pietroski’s (2009, p. 133) claim that, semantically, “concatenation is an instruction to conjoin monadic concepts, while labeling provides a vehicle for invoking thematic concepts, as indicated by the relevant labels.” Dyadic concepts like INTERNAL(E,X) can be introduced by labels, with lexical items delivering the required information to fill in the thematic content.

**FIGURE 3:**
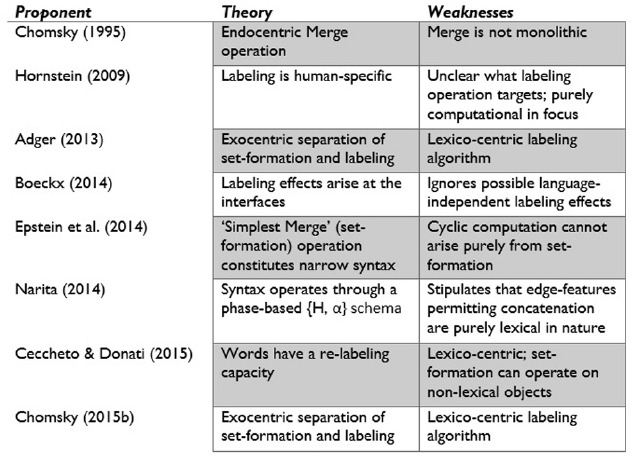
**Theories of the human cognome, specifically relating to language**.

Labeling may also have yielded the vast categorization abilities of humans, perhaps even of the type-token sort (Jackendoff, 2007, p. 106). Hornstein (2009, p. 58), for instance, has noted that “labeling incorporates what Boeckx (2006) describes as rigid categorization (dominance by type) and so it is not surprising that when labeling emerges so too does this cognitive ability.” Nevertheless, as mentioned, labeling is still predominantly seen as a side effect of Merge. A recent monograph by Citko (2011) bears the title *Symmetry in Syntax: Merge*, *Move*, and *Labels*—the noticeable “s” pluralising “label” reflects this tendency to relegate the labeling operation itself and focus on its products. The account I am outlining here goes beyond Hornstein’s theory by placing labeling at the centre of an ethologically unique model of organic computational capacities (see Figure 3 for a summary of the major theories of Merge and labeling).

Lastly, by lending neurobiological validity to the separation of lexicalisation, set-formation, labeling and cyclic transfer, recent work by Ramirez et al. (2015) supports the view that labeling alone is not sufficient for cyclicity. Instead, a labeling operation leading to a transfer operation to *both* the CI and SM interfaces (unlike in Campbell’s monkeys, which exhibit transfer purely to the former, and birdsong to the latter) would be necessary for the labeling effects found in human cognition. These cyclic computations would perhaps be operationalised at different points, as the decomposition of Spell-Out mentioned above would imply.

### Roots and Categories

Removing the long-standing Distinctness Condition on Merge, Adger (2013) demonstrates that if α and β are distinct, or one part of the other, or identical, these three logical possibilities give rise to concatenation, movement and Self Merge. If α and β are identical, Self Merge would yield {α,α} = {α}. As Boeckx (2014, p. 47) summarizes, Self Merge “in effect turns one of the atomic elements into a phrase, allowing the merger of two atomic lexical items to comply with … the H-α schema.” Following the lead of Distributed Morphology, if we assume that every LI has a “root” (Marantz, 1997) then every sentence is constructed by roots being Self Merged with themselves. This occurs because bare roots cannot be “seen” by the labeling algorithm. The root √*book* would Self Merge to create √*book*, which would then be labeled by a universal sequence of functional categories, yielding _N_√*book*. By showing that labeling yields thematic concepts while concatenation yields simple monadic concepts, Hornstein and Pietroski (2009, p. 126) explain that the verb *stab* results from concatenating the root √*stab* with a verbal element which labels the structure, [{_*V*_√ stab^*V*}]. Roots cannot be merged with other syntactic objects, but require Self Merge, whilst labeling would be impossible in pure Self Merge, since unary branching structures cannot be interpreted by the external systems. This leads to what we could call the Root-XP Constraint:

(10) *Root-XP Constraint*

*{√R XP}

I assume that functional categories are not LIs as they are in Bare Phrase Structure, which betrays its “bare” nature by adding further endocentricity through claiming these categories are inherently linked to LIs. Functional categories arise via Self Merge of roots, and instead of the classical division between functional and lexical LIs, Adger proposes a Root Lexicon and a Category Lexicon:

(11) RLex = {√1,…, √n}, the set of LIs (roots)

CLex = {1_1_,…, 1_n_}, the set of category labels

RLex plus Concatenate (κ) yields hierarchical structure, and CLex provides the labels for these structures. This formulation rightly departs from the Probing Algorithm of Ceccheto and Donati (2010), which states that only the Probe (Chomsky, 2001b) in a Merge operation can provide the label (see Murphy, 2015c for discussion). RLex is close to classical conceptions of the “lexicon” or the Type L system of the IH (Miyagawa et al., 2014), while CLex resembles the universal base (Cinque, 1999).

### Syntax Without Ethology is Lame

In response to the question of what led to the Upper Paleolithic Revolution (Bar-Yosef, 2002), Chomsky responds “Merge,” Ramachandran (2011) responds “Mirror neurons,” Jackendoff (2007) responds “Parallel Architecture,” but I think the evidence increasingly suggests that the answer is “Label.” Many current models ignore this operation and stick to investigations of concatenation. To take a recent case, the IH sidelines labeling and claims that language is simply the result of the convergence of two finite-state systems, the misleadingly titled “L(exical) structure” (observed in bee dances and primate calls) and “E(expression) structure” (observed in birdsong), claimed to be brought about by the arrival of Merge. Although the general picture of the computational system interfacing with other systems is doubtless correct, by sticking solely to this observation IH only succeeds in formalizing a platitude, despite whatever positive impact its persuasive rejection of Neo-Darwinian gradualism may have on the field. Decomposing the interfaces into finite-state systems has some value, but IH is oddly silent on the *nature* of the computational system which integrated these pre-adapted systems; Nóbrega and Miyagawa (2015) only comment that Merge gave rise to “a single non-finite generative-engine capable of yielding any sort of linguistic object,” with the cautiously ambiguous nature of the term “non-finite” and their lack of ethological range noticeable.

### Ethology Without Syntax is Blind

It was noted above that significant advances will be delayed so long as linguists continue to sideline ethologists, but inquiry will also suffer from linguists sidelining other linguists; namely, those concerned with mathematical description and formalism. Even Zuidema (2013b, p. 181), in his careful summary of artificial grammar learning, notes only that human language is “guided by a system of semantic and syntactic categories and rules,” not going into any specifics which could be analyzed alongside proposals in the animal cognition literature. Instead, he proposes that the evolution of language reduces to the emergence of “hierarchical compositionality” (Zuidema, 2013b, p. 186), again failing to elaborate or produce a fine-grained definition. Such a precise account at the computational level is vital in order to differentiate between cognitive capacities across the ethological spectrum, and failing to produce such an account often leads to misunderstanding and premature, even grand statements of theoretical coherence and success. Consider, for instance, Zuberbühler’s (2015) review of the “linguistic capacity of non-human animals,” which claims that “the origin of language is the result of multiple gradual transitions from earlier forms of primate-like communication and social cognition.” Zuberbühler reaches this conclusion through examining only the performance systems (e.g., vocal tract control) and extra-linguistic cognitive systems (e.g., awareness of audiences during externalization) of non-humans, sidelining completely the computational competence system.

The science of language will therefore be stymied so long as linguists continue to sideline ethologists, and ethologists continue to embrace nebulous and inadequate conceptions of syntax. Indeed, three prominent zoological researchers (Lemasson et al., 2013, p. 183) describe it as the study of “how independent meaningful units are combined into more complex utterances,” failing to elaborate beyond this. Without a theory of the objects *language* and *grammar*—the goal of theoretical linguistics—it is impossible to assess the merits of attributing such capacities to animals.

### Narrow Labeling

If non-humans are ultimately shown by ethologists to have context-sensitive grammars, this would imply that their grammars are characterisable by a non-terminal alphabet, employing “labels.” This would seem to disprove the human-unique theory of labeling developed here. However, to equate non-terminals with labels is not an *a priori* step (contra Narita, 2014, p. 18), for we would have to premise this by claiming that the non-terminal will be targeted by a subsequent operation and then interpreted, otherwise the label would not be needed. Labels, Epstein et al. (2014, p. 472) comment, are “arguably a natural requirement necessary for CI interpretation.” Further to this, in natural language labels employ narrowly linguistic functional categories (Gallego, 2010), and so even if ξ were not human-unique, the elements it operates on certainly are. Considering evidence that animals such as pigeons can “chunk” information (Terrace, 2001), forming a type of non-terminal atom of computation, we can comment that labels and non-terminals should not necessarily be equated, and that cyclic transfer to *both* interfaces is required for labeling effects to arise.

I will return to this tension below, but for now another concern can be raised: If I am endorsing exocentric labeling (i.e., the sort of labels needed in Type 1 and 2 grammars), how can we still speak of endocentricity? The answer lies in acknowledging that (i) the cognitive features attributed to endocentricity (e.g., prominence), and the syntactic notions traditionally tied to it (e.g., X-bar-theoretic heads), are by no means one and the same, and (ii) exocentric labeling can give rise to such representations. Endocentricity is not the only phenomenon requiring labels: interface phenomena like prosodic domains (Samuels, 2011), islands (Boeckx, 2003), and incorporation (Baker, 1988) also require them. Under an exocentric system such as the one adopted here, labeling is not “projection” at all, but rather what we could call “injection,” assigning to roots a category label. Hornstein and Pietroski (2009, p. 116) argue further that “the meaning of any complex expression is the meaning of a labeled concatenation.” If labeling effects are ultimately found outside language, perhaps in the visual domain, then further investigation will be needed to establish whether the application of ξ is free and lacking a “purposeful” existence in the way that applications of Simplest Merge are now thought to be (Epstein et al., 2014, p. 463).

The distinctive mark of language may not be its “infinite use of finite means,” then, but rather the way it breaks the bounds of semantic monotonicity by projecting new categories upon the concatenation of two syntactic objects, giving us different kinds of “things” to think about. We can continue to term the outputs of ξ “projected” structures so long as we recognize the descriptive status of the term, in a similar way that syntacticians still speak of “phrase structure” despite the elimination of this component many years ago. Language is also doubtless recursive, but what Hinzen and Sheehan (2013, p. 101) call its “substantive content” is found not in recursion (contra Chomsky, 2014), but rather in the way ξ provides the means to introduce to atomic roots functional structures which serve as the hosts of CI notions like predicate and argument.

### The Labeling Algorithm

What has been presented so far begs an important question, one of the most central in modern linguistics. Adger (2013) names this the Labeling Problem: Is there a unified labeling algorithm that applies to all syntactic configurations? To answer this question it is useful to refer to what Adger (2013, p. 21) calls a Universal Extended Projection of a category C, a sequence of labels drawn from CLex (1_s_,…,1_t_), 1_s_ being the Start Label and 1_t_ the Terminal Label. Adger states the binary Cartesian product of CLex as a set of label transition functions (LTFs), denoted by Λ. This yields an algorithm which applies category labels to syntactic objects in a constrained, sequential fashion:

(12) Λ = CLex × CLex = { <N,C1> , <N,N> , <C1,N> , <C1,C1> , <N,Num> ,…}

It follows that “for any particular (I-)language, some subset of Λ will exist and will define, for that language, the particular extended projections available … Part of the acquisition process is determining what the content of Λ is” (Adger, 2013, p. 21). ξ takes an unlabelled object as its argument and a category is assigned to it via cyclic Transfer to the interfaces. The labeling algorithm must furthermore apply at this derivational step, since structures cannot differ at the two interfaces, being a v*P at CI but a CP at SM (Chomsky, 2015b). Each I-language amounts to a subset of possible LTFs. There are thus no functional heads, only functional structures admitted by Λ. In addition, this reveals the stipulative nature of the thematic domain in syntax, something which has typically not been appreciated by minimalist philosophers (for instance, Mukherji, 2010).

With this Λ-system, the trouble over specifiers, complements, and the “problems of projection” (Chomsky, 2013) fade away. But on these Λ-system grounds, as Svenonius (2012) would argue, labeling is actually a finite state operation, since memory is not needed to label the object under manipulation at any derivational point if labels are not recognized but drawn from a set. It is consequently possible to simply stipulate CLex/RLex relations or overgenerate until a convergent result is reached. Instead of stipulating a syntax-external CLex (where, after all, is such a system embedded in the cognitive architecture, and where does it come from?), categories rather emerge at the interfaces through what I will call the *Labeling Assembly* (LA), produced through the interaction of (i) general cognitive constraints, (ii) the CI-system, (iii) the cognome (ς_H_) and (iv) the lexicon. Hence labels are not purely recognized at the semantic interface (as in Gallego, 2010 and the more dubious account in Krivochen, Forthcoming). This does not, however, imply that labels are purely interface phenomena, since the operation which constructs objects to be assigned labels from the LA is independent of both concatenation and transfer. Functional categories are composed of interpretable properties such as force, finiteness and modality; things which are likely language-independent aspects of cognition. Note also that the LA is not an independent system, but rather a convenient way of denoting the interaction of the above four factors. In addition, for reasons discussed below, I will replace Adger’s reliance on fully-fledged roots via RLex with Boeckx’s (2014, p. 27) suggestion that the objects of narrow syntax are *flat* and *atomic*, what he terms “lexical precursor cells” (LPCs). We can call this modified, reduced lexicon the Precursor Lexicon, denoted by *_p_*LEX. Additionally, if we consider Poeppel’s (2012) granularity mismatch problem, Adger’s claim that the labeling algorithm purely applies to roots is troubling; I would alternatively suggest that it applies instead to LPCs, objects which are less language-specific.

Since I have replaced CLex with the LA and RLex with *_p_*LEX, the formal properties Adger discusses should consequently be reconsidered from this perspective. Modifying Adger’s (2013, p. 22) theory for the system pursued here, where α and β are syntactic objects, the unified labeling function is as follows:

(13) a. Transition Labeling: If α,β ∊ γ, then Label(γ) = some LPC∊*_p_*LEX, such that there are (possibly non-distinct) f and g ∊ Λ such that f[Label(α)] = g[Label(β)] = LPC

b. Lexical Precursor Cell Labeling: Label ({√x}) = some LPC ∊ {N,V,A}

Proceeding within this framework, the label of a syntactic object is “dependent on (but not identical to) the label of both its subconstituents” (ibid.). All derivations begin with the root, say √*chair*, being taken from *_p_*LEX by Select, Self Merging to create {√*chair*}, then being labeled _N_({√*chair*}) by Λ, which in this case would project “up” to D, given the following Λ for English (where C1 is the category born by a classified noun):

(14) Λ = { <N,C1> , <C1,Num> , <Num,D> …}

This labeling algorithm would produce the following extended projection: [D[Num[C1[N[√*chair*]]]]]. Assumptions of simplicity lead me to qualify that the ordering of labels is likely not part of universal grammar (UG), but rather emerges from the complexity of different computational orderings.

### Formalizing ξ

Since External Merge is a context-free operation, creating γ from any given α and β in a derivational space, it is available for a Push-Down Automata and permits centre-embedding (“true”) recursion. As mentioned, the Finite State Automata required in the grammars of, for instance, Bengalese finches, would be capable of only tail recursion. Yet if we endorse the decomposition of Merge into κ and ξ seen above, then in Section “Simplest Merge and Labeling,” then ξ requires an algorithm to analyze α and β to detect categorical features. Hence ξ must be MCS—demanding an extended Push-Down Automata (PDA+) to implement (Rychnovský, 2009)—if it is also requires to search an object δ and extract/copy α ∈ δ and concatenate it to δ, a case of reprojection (Surányi, 2008). The labeling effects seen in word movement or “Internal Merge” (Chomsky, 2001a; Stabler, 2011), the concatenation of an object which is part of another, require “a more developed procedural memory system” than pure concatenation or “External Merge” (Bolender et al., 2008, p. 138, contra Chomsky, 2013, p. 41). Thus while Merge in Chomsky’s (2013) sense is non-Markovian, the operation of κ we extract from it is. It follows that the only kind of behavioral evidence ethologists could use to argue in favor of an MCS ξ operation in non-humans would need to exhibit unbounded applications of concatenation, with atoms (e.g., song or call units) forming constructed objects which could then be used as atoms themselves, having been given a syntactic status independent of their parts.

Svenonius (2012) additionally claims that features are combined using two mechanisms. The first is “the finite state network, which allows feature bundling in featurally complex heads. The other is Merge, which allows embedding of one category inside another.” But embedding via κ is achieved only in the event of ξ, and while feature bundling can be achieved in a finite state network, the searching of lexical items and deployment of external memory seen with ξ extends beyond the feature bundling discussed by Svenonius. Further, instead of adopting Svenonius’s Bundle operation, which combines features into lexical items, I would argue that κ is, to borrow Berwick’s phrase, “all you need” to construct feature bundles. Only then is ξ used to attribute to merged sets a syntactic status determined by emergent properties of the embedding of ς_H_ within the external systems, since bundling features is not computationally equivalent to labeling, contra Boeckx (2014, p. 115). Functional hierarchies must therefore be emergent at the LA; a case of third factor effects upon ς_H_ arising via general cognitive mechanisms yet to be determined. The Λ-system remains, then, but with this important qualification regarding its syntax- and interface-external origin.

To briefly illustrate the workings of the proposed system: LPCs are taken from *_p_*LEX, Self Merged, concatenated to form a set, and labeled via the LA and Λ at the point of transfer to the interfaces.

### Architecture

Synthesizing what we have reviewed above, the basic architecture of our system is as follows:

(15) a. ς_*H*_

b. *_p_*LEX

c. Interfaces

When Pietroski (Forthcoming) speculates that the reason why *lamb*, *Venice*, and other lexical items are so combinable is because the acquisition of a lexicon allowed humans to efface many typological distinctions, we can respond by pointing to the labeling of flat and atomic roots from *_p_*LEX via the LA. Labeling allows the brain to create items which can be freely deployed at any time, partly independent of perception. When Λ, operating through the LA, categorizes a root as either N or V, ultimately yielding *a swim* or *to swim*, it achieves a level of cognitive flexibility and innovative world-making in Chomsky’s (1972) sense which nothing else in the natural world seems capable of emulating.

With the architectural framework clear, how can we now evaluate the cognome? Transfer, Spell-Out, and Interpret, though typologically distinct to humans, are most likely generic information-transmitting operations, realized in other species. The same applies to Check. The major operations of phonology, Align and Wrap (Scheer, 2011, p. 390), though operating within prosodic and syntactic phrase boundaries, may also reduce to the kind of segmentation demonstrated by Bengalese finches and other avian species. Collier et al. (2014) review the literature on animal call sequences and conclude that phonology in non-human vocal systems is rare, unlike basic combinatorics, suggesting that the particular operations of human phonology evolved after narrow syntax. As we have seen, κ is an ancient operations rooted in various forms of animal cognition. That leaves ξ. I would argue from this that ξ is the human innovation absent in other species. We now return to the ethological consequences which immediately follow from this.

## Computational Ethology

This final section will expand the syntactic considerations found above by relating biolinguistic concerns to ethological concerns. Suggestions to the ethological studies explored in Section “Approaching Computational Ethology: The Evidence.” Suggestions for testing the Labeling Hypothesis are also put forward.

### Weak Continuity

If syntax can “carve out” a wide variety of semantic phenomena in human language, with κ and ξ giving rise to semantic compositionality, a (currently unanswered) question arises: Do the computational systems of other species also perform such a role? “[O]ur knowledge of the possible conceptual-intentional processes underlying bird vocalizations is almost non-existent,” caution four prominent researchers (Langus et al., 2013, p. 242). At the same time, the CI systems of non-humans plainly operate with cognomes which can carry out computations impossible for humans. For birds and bees, “The local solar ephemeris is a spatio-temporally contingent aspect of experience; it varies as a function of both latitude and time of year, so it must be learned” (Gallistel, 2011, p. 254). These should be the guiding concerns of what I will call Computational Ethology (which can be seen as a branch of what Benítez-Burraco and Boeckx (2014) call “comparative biolinguistics”), a field aimed to be well embedded within the interdisciplinary explorations of the life sciences. Certain linguists have also taken note of such concerns; Bolender et al. (2008, p. 132) recommend that, “So far as possible, seek explanations of uniquely human concepts in terms of syntactic computations.” We can adapt this maxim to Computational Ethology and propose the Carving Guideline:

(16) *Carving Guideline*:

Seek explanations of non-human conceptual systems in terms of the application of operations from the cognome.

**FIGURE 4:**
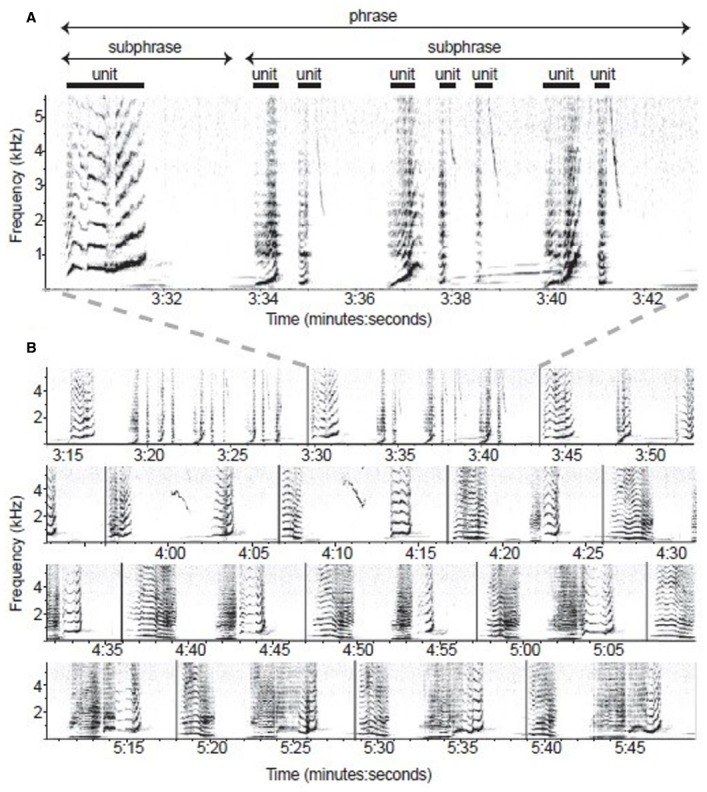
**Spectrographic model of humpback whale song recorded at Isla Socorro, Mexico, on 27 March 2006 (from Cholewiak et al., 2013, p. E318)**. Note that multiple sub-phrases group into a phrase, which can vary in their temporal and spectral characteristics, leading to the suggestion that “humpback *phrases* and bird *songs* are analogous” (E324; compare with **Figure 1). (A)** One phrase composed of two sub-phrases. **(B)** Multiple phrase types exhibited throughout 155s of song sequence. Phrases are defined through vertical lines.

Given that cyclic transfer of labeled structures to the CI interface may be unique to humans, the silence of many ethologists when it comes to issues of semantics (and not just meaningless symbol sequences processed by the SM interface) should be of great concern. The songs of birds (Berwick et al., 2011; Slater, 2012) and cetaceans (Cholewiak et al., 2013, see Figure 4) are distinct from other forms of non-human externalization in displaying hierarchical structures. While there is no evidence of compositional semantics or recursion, whale song has been shown to be “built up from themes, consisting of phrases, consisting of units, in turn built up from subunits. Hence, whale song might rightly be characterized as hierarchical” (Zuidema, 2013b, p. 180).

Suzuki et al. (2006) demonstrated the existence of a strong structural constraint in humpback whale (*Megaptera novaeangliae*) songs which cannot be represented in Markovian terms. But, as with birdsong, these combinatorial abilities are independent of conceptual structures. Hierarchy, though limited, is present in the social cognition of spotted hyenas (Holekamp et al., 2007). The songs of starlings, nightingales and Bengalese finches contain elements which may be followed by numerous others, and the structure of the song is determined by probabilistic rules between a finite number of states. Indeed the “structural flexibility” of birdsong may “suggest that the computations necessary for sequencing the song elements are independent of the sensorimotor representations over which they operate” (Langus et al., 2013, p. 232).

Taking stock, with labeling being unique to humans the cognome of a Bengalese finch would perhaps be (minimally) as follows:

(17) ς_TAENIOPYGIA GUTTATA_ = {κ, *χ*}

This cognome can additionally be attributed to Campbell’s monkeys, based on what we saw in Section “Approaching Computational Ethology: The Evidence” based on the evidence reviewed. The finch would also employ Transfer operations, but the above appear to be the bare necessities, with the finch’s stock of vocalization types permitting numerous tokens of these vocalizations to be repeatedly externalized, hence some form of Copy operation would conceivably be involved. Animal signals do not display anything related to agreement relations or grammatical types, and given the discontinuities between human syntax and such signals it becomes untenable to support either a protolanguage hypothesis or what I will call a *Strong Continuity Hypothesis*, invoking gestures, music or theory of mind as language precursors.

(18) *Strong Continuity Hypothesis*: There are no novel operations employed by ς_H_, and it is only certain human-specific physiological and behavioral traits (e.g., bipedalism, long infant dependency), along with a specific combination of cognitive faculties found in other species (e.g., theory of mind, imitation) which are the distinguishing features of humans.

The related claim that language evolved from a single precursor has been criticized from a number of fronts (see Hauser et al., 2014). Why imitation and self-awareness are relevant to the inheritance of φ-features is never made clear by proponents of strong continuity (e.g., Gibson, 2013, p. 201–211). Diverging from this line of inquiry, Hinzen and Sheehan (2013, p. 73) conclude that many animals perform tasks that “require the computations of values for variables in such a way that a homomorphism exists between the internal computations and the perceived structure of external reality.” Humans managed to “obtain internal address systems (roots) for their representations of environmental features, which can be used to symbolically refer to the world,” a drastic representational change which supports a *Weak Continuity Hypothesis*:

(19) *Weak Continuity Hypothesis*:

Novelty of computational capacity, along with certain physiological and behavioral traits, are the distinguishing features of humans, and only limited cases of continuity exist between the mental representations generated by φ_H_ and non-human CI systems due to the lack of cyclically transferred labeled structures in the latter.

This position can be defended by referring back to the above discussion concerning the cyclic transfer of labeled structures to both interfaces, which appears to be unique to humans. The Weak Continuity Hypothesis, and the concerns of Computational Ethology more generally, become even more apparent when we note the rapid shift in priorities in much of contemporary phonological theory, which has firstly moved from asking “Do songbirds have recursion?” to “Do songbirds have labels?,” and secondly has begun to inquire into whether human phonology employs labels (Van Oostendorp, 2013). Discussing this final topic will take us too far afield, but we can at least conclude that labeling appears to have re-formatted mental life by providing what Boeckx (2010, p. 128) would call a “universal currency” carrying out cross-modular transactions between the “specific vocabulary” (Chierchia, 2010, p. 166) of “core knowledge systems” such as OBJECT MECHANICS and NATURAL GEOMETRY (Kinzler and Spelke, 2007; Carey, 2009). Lexicalisation “puts all concepts on a par, making them all accessible in the workspace” (Boeckx, 2012, p. 49), “going beyond modular boundaries” (Boeckx and Benitez-Burraco, 2014, p. 4), becoming the LPCs of *_p_*LEX. As Ursini (2011, p. 215) phrases it, “language represents a “neutral” logical space, a model of knowledge representation in which different concepts can be freely combined.”

I would like to suggest that Computational Ethology, by exploring the capacities of various cognomes across the ethological spectrum, has the potential to act as a powerful tool for comparative biolinguistics, with the major obstacles currently being methodological and theoretical/computational. For instance, in addition to the problems raised above concerning definitions of primitive operations, there is also the likelihood that formal language hierarchies are inadequate in evaluating a substantial range of cognitive capacities (see the scattered range of natural language patterns in Figure 2), no matter how precisely they map onto particular embedding and nesting procedures. Computational Ethology should begin to move away from formal language hierarchies, useful though they may have been, toward the kind of finer-grained analysis which theoretical linguistics can deliver. The Chomsky Hierarchy is ultimately too generic and “weak,” since various cognitive methods can yield a single MCS structure, not just the human labeling algorithm. Different aspects of phonology and syntax also fall into different subregions of the hierarchy, making claims about artificial grammar learning yielding insights into monolithic concepts like “syntax” inherently misleading.

### Approaching the Labeling Hypothesis

Having presented the Labeling Hypothesis, along with the broader Weak Continuity Hypothesis, concerns immediately arise about how to test the predictions of a label-only theory of language evolution. Perhaps the most direct evidence in support of the Labeling Hypothesis would arrive in the form of data revealing either (i) a lack of cyclicity not just to the SM interface, but also the CI interface, or (ii) a lack of cognitive effects arising from the form of trivial tail recursion found in, for instance, birdsong. For instance, returning to the discussion of wild mountain gorillas, testing whether nettle preparation involves hierarchical planning or a looped routine requires isolating the (proposed) hierarchical procedure itself, abstracting away from the initial memorized routines of finding a nettle patch. If the procedure involved in stripping stems and tearing off petioles is indeed hierarchical, then the gorillas would also be able to perform an unrelated task with the same number of steps and, crucially, the same form of hierarchy at a different point in the procedure (perhaps at the second, and not third, step). This would control for the effects of short term memory capacity, and if the new task is unrelated both to nettle preparation and the gorilla’s lifestyle then performing it successfully, given the inclusion of a hierarchical procedure, would suggest that the gorillas had not memorized a looped routine but are in fact utilizing their set-forming and chunking capacities in novel circumstances. Further behavioral investigations could test this on other subspecies of gorilla, considering the limitations of conducting experiments on the endangered mountain gorillas (see also Dawkins, 1976 for discussion of the efficiency of hierarchical organizations of common subroutines).

Effective ways of testing the Labeling Hypothesis could also come from eliminating many of the current obstacles found in ethological experiments. For instance, numerous studies of birdsong include redundant variation in syllable elements (e.g., Gentner et al., 2006), forcing the two unrelated tasks of syllable categorisation and sequence learning to conflict. Ignoring this introduces confounds and leads to premature conclusions about both the counting and sequencing capacities of birds. Decomposing tasks is equally as important as decomposing the computational operations animals are claimed to perform. In domains outside of linguistics, however, a focus on computational issues is sorely lacking. Attending to these concerns could potentially refine discussions of particular ethological and psychological concepts. For instance, it has been noted (Terrace, 2002, p. 25) that an operational definition of chunking is largely absent from a great deal of research. Embedding such opaque constructs within a framework of syntactic combinatorics may result in the limits and range of such capacities being exposed; much like how decades of computational inquiry into grammatical phenomena led to an understanding of certain generic properties of language such as minimal search principles (Narita, 2014; Chomsky, 2015a; Larson, 2015).

**FIGURE 5:**
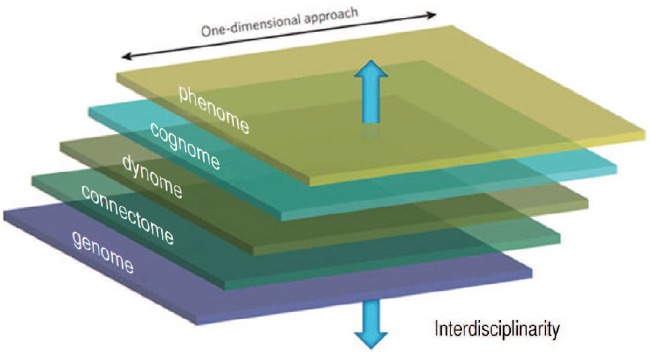
**One-dimensional and multi-dimensional approaches to the mind/brain contrasted (from Boeckx and Theofanopoulou, 2014, p. 406)**.

The perspective of Computational Ethology advocated presently also ought to be framed in the context of a broader investigation linking the cognome to the dynome [the study of brain dynamics and oscillations (Kopell et al., 2014)], the connectome [the set of neural connections in a given nervous system (Sporns et al., 2005)], and ultimately the genome, as advocated in Boeckx and Theofanopoulou (2014; see Figure 5). The priorities of each of these disciplines needs to be considered by the computational ethologist and the linguist working one way or another within the Decompositionalist Project (Murphy, 2015c, see also Embick and Poeppel, 2015).

Considering briefly the genome, is has been proposed that 1,241 primate-specific genes exist (Zhang et al., 2011), 280 of which are human-specific. 54% of these human-specific genes are upregulated in a brain area implicated in higher cognition, the prefrontal cortex. These new genes are “much more likely to be involved in gene regulation” (Diller and Cann, 2013, p. 256). Consequently, it is not unlikely that the mutation of some regulatory gene reorganized the neuronal populations in the neocortex and the computational properties concomitant with it, partly contributing in the system pursued here to the emergence of ξ, a genuine evolutionary novelty. This position seems reasonable given recent evidence that the complex syntax of Bengalese finch songs developed from simple neurological changes (Katahira et al., 2013). Although given the level of regulatory complexity identified by Chakravarti (2011), it is more likely that ξ emerged after the mutation of multiple regulatory genes acting in concert, and not a singular mutational event as often claimed by minimalists (e.g., Chomsky, 2010): “Genes and their products almost never act alone, but in networks with other genes and proteins and in [the] context of the environment” (Chakravarti, 2011, p. 15). Of relevance here is the finding that human evolution has slowed down, often called the “hominoid slowdown”: “[R]ates of occurrence of de novo mutations decreased as enhanced DNA repair mechanisms and larger generation times evolved” (Goodman, 1985, p. 10). Hominoids appear to have reached a certain mesa of complexity, with only slight tuning yielding novel benefits. In summary, a slight epigenetic change, call it the “Small Bang,” could have produced an alteration in the human computational system yielding ξ.

**FIGURE 6:**
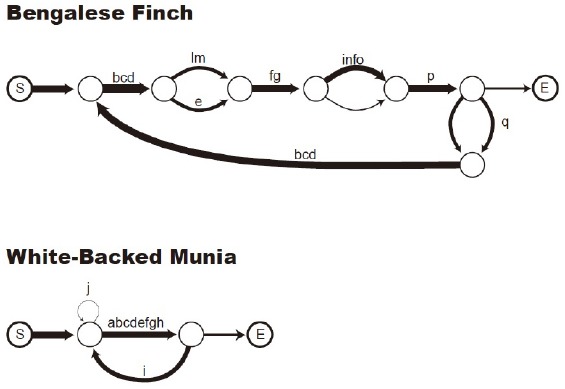
**Transition diagram of a domesticated Bengalese finch song (upper) and a wild white-rumped munia song (lower)**. “S” indicates the start of a song, “E” indicates the end (from Okanoya, 2012, p. 48).

A separate question now arises concerning when the Small Bang took place. Putting aside precise dates, and assuming that anatomically modern humans emerged around 150–200 kya, it appears that complex forms of symbolic representation did not begin until 60–100 kya (Hurford, 2011). This is also the time period correlating with new migration patterns (Mellars, 2006), leading to the possibility that properties of the environment acted as release factors for the labeling capacity, as they also appear to do for the singing capacities of genetically identical finches subject to distinct environmental upbringings (Okanoya, 2012, see Figure 6). Encountering new forms of social organization and environments may have served to prompt the basic combinatorics of mammalian cognition and encourage novel forms of (ultimately cyclic) conceptual combination. Bolender (2007) has suggested along these lines that an increased human population, leading to a greater complexity of inter-group communication, acted as a trigger for the use of word movement, hitherto dormant. If this is correct, then investigating syntactic phenomena from a purely computational perspective, not considering the influence of the development and emergence of the phenotype, would be missing a crucial part of any explanatory account.

I would also like to raise a final prospect for Computational Ethology in relation to the dynome. Given that the hierarchy of brain oscillations appears to have remained extraordinarily preserved during mammalian evolution (Buzsáki et al., 2013), the construction of a new substitute for the flawed Chomsky Hierarchy, which makes brain rhythms and computational operations like set-formation and labeling commensurable, is an urgent challenge. Brain rhythms can also enhance understanding of information chunking (Giraud and Poeppel, 2012), and could therefore act as a bridge between neurobiology, ethology and the algorithmic and computational levels of syntactic theory. The research of what we could call “Rhythmic Syntax” could lead to an expansion of the concerns of ethologists and the priorities of linguists, many of whom pay lip-service to how language can act as a window into the brain but rarely step outside the confines of debates about, for instance, ellipsis and binding theory. Any account of syntactic phenomena will inevitably be deficient if it fails to consider the release factors of mental capacities and the brain rhythms which form and constrain computation. Research on this front may well prove stimulating and fruitful (McCormick et al., 2015; Murphy, 2015a). In short, Rhythmic Syntax will likely allow us to “see” labeling effects more clearly than the forms of behavioral and neuroimaging data ethologists and neurolinguists are used to. Through placing the brain at the centre of inquiry, both Computational Ethology and the Decompositionalist Project are in turn aligned closer to the comparative biolinguistics advocated by Benítez-Burraco and Boeckx (2014) than they are to generative grammar and its recent (though illuminating) approaches to language as a system of minimal computation. These debates should consequently not be confined to minimalist quarters.

## Conclusion

The Labeling Hypothesis proposed here, in concert with the Weak Continuity Hypothesis, lead to new directions for ethological and syntactic inquiry into the computational capacities of humans and non-humans. The field of Computational Ethology which proposes to test these hypotheses has yet to be undertaken to any serious degree, with only a small number of researchers investigating the cognomes of the great apes and a small number of avian and other species. But if the task of the linguist is to explore the computational properties of mental structures in an effort for these principles to be employed as the goals of neurobiology, then it is hard to see how these programs could fail to be worthwhile. It is additionally my hope that this contribution will lead to the strengthening of the computational rigor of ethological inquiry, and to further interdisciplinary research into the nature of the Small Bang. GPS monitoring, bioacoustic analysis and telemetric recordings can enhance observation, but substantial theoretical advances will only arrive if the computational studies of syntacticians are acknowledged by a wider audience.

### Conflict of Interest Statement

The author declares that the research was conducted in the absence of any commercial or financial relationships that could be construed as a potential conflict of interest.
